# Association between radiographic equine distal phalanx characteristics and absence, presence and type of horseshoes

**DOI:** 10.3389/fvets.2025.1598038

**Published:** 2025-07-25

**Authors:** Lisa Henrietta Ennsmann, Theresia Franziska Licka

**Affiliations:** ^1^Department of Companion Animals and Horses, University Clinic for Horses, University of Veterinary Medicine, Vienna, Austria; ^2^Department of Veterinary Clinical Sciences, Large Animal Hospital, Easter Bush Veterinary Centre, Easter Bush, Royal (Dick) School of Veterinary Studies, The University of Edinburgh, Roslin, Midlothian, United Kingdom

**Keywords:** horse, distal phalanx, radiology, horseshoe, clips

## Abstract

Most horses are used with horseshoes additionally supported by either dorsoabaxial or dorsal clips. The effects of such clips on bone density and shape of the distal phalanx are currently unclear. The aim of this study was to identify correlations between density and shape of the distal phalanx, comparing front hooves unshod or shod with standard shoes either with two dorsoabaxial clips or with a single dorsal clip. Researchers analyzed Oxspring radiographs of either the left or right front hoof from warmblood horses (*n* = 132) and ponies (*n* = 43) aged 3–28 years. The evaluation focused on distal phalanx density at the margo solearis, particularly at three locations corresponding to the clip positions: dorsomedial, dorsal, and dorsolateral. The study examined horse related variables such as age, breed, use, and shoeing type in relation to density parameters, presence of a crena marginalis solearis, an anatomical variation that is an indentation dorsal on the margo solearis, and the shape of the distal phalanx. Distal phalanges of hooves shod with dorsoabaxial clips showed a significantly (*p* < 0.001) lower width to length ratio (median 1.31, minimum 0.70, maximum 1.66) compared to those with a single dorsal clip (median 1.40, minimum 0.89, maximum 1.75). The width to length ratio of unshod hooves (median 1.37, minimum 0.80, maximum 1.82) was not significantly different from both groups of shod hooves. The results of this study should be considered when selecting horseshoes for equids.

## 1 Introduction

Humans first shod horses to protect hooves from excessive wear (1). Later, metal clips, semicircular metal protrusions from the shoe that conform to the dorsal or dorsoabaxial hoof wall, were added for additional support (2). The distal phalanx (P3 or coffin bone) forms the hoof’s structural foundation and is critical for equine locomotion (2). It connects to the horn capsule via the laminar interface, enabling efficient load distribution (3). Since the advent of radiography, hoof and P3 morphology have informed shoeing practices (4). Farriery and trimming significantly alter hoof shape (5), which can change notably within 7 weeks of either shoeing or barefoot management (5). Posture and nutrition also affect hoof horn quality and shape (6), thereby influencing P3 morphology (7). Hoof shape is a key determinant of locomotor function in horses (7).

Throughout the 20th century, various methods were used to study shoeing effects on equine performance. Shoe mass increases distal limb mechanical load (8), and standard shoeing increases stride length and joint flexion compared to unshod horses (9). Also, increased stride duration and stride length in trot was noted (9). Aluminum shoes yield a larger minimum carpal angle and lower maximum hoof height than steel shoes (10). Force plate studies documented limb loading and movement, with shod horses showing greater maximum vertical ground reaction forces (11). Pressure plates demonstrate that shoes with a wider toe increase toe contact area and reduce vertical pressure in the toe region (12).

Clips improve horseshoe stability and reduce nail strain (13), this is especially important if the traction of the shoe is enhanced using studs, pins or borium. Clip configuration varies by discipline: dressage horses are typically shod with one dorsal clip on forelimbs and two dorsoabaxial clips on hindlimbs, while on draft and racehorses often a single dorsal clip is used on all hooves (14). In racehorses, dorsal hind clips prevent backward displacement and protect high-power transmission zones (15).

Shoeing alters hoof biomechanics, shifting the center of pressure medially during early stance (13). Finite element models reveal that dorsoabaxial clips exert maximal pressure on the hoof wall by restricting normal hoof expansion at clip positions during loading (14, 15). These clips limit hoof mechanism (14), potentially affecting growth and condition (16). Dorsoabaxial clips are occasionally used on front hooves despite impairing hoof mechanism (14, 15). These clips lead to narrower hooves and they can shift the breakover point and reduce hoof expansion (17–20), though effects on the distal phalanx have not yet been considered. Nail placement also influences mechanics, with heel-proximal nails restricting hoof expansion (21).

Thermography revealed cooler soles and reduced blood flow in shod hooves compared to unshod hooves after 8 weeks (22). As in all bones, the forces sustained also affect the osseous quality of the distal phalanx. Bone is constantly turned over, which involves modeling and remodeling mediated by osteoblasts, osteoclasts, and osteocytes, maintaining bone balance (23). A shift toward resorption results in bone loss (24), and excessive strain may weaken the bone potentially leading to microdamage/stress fractures (25). Pathological changes in the distal phalanx occur with conditions such as laminitis, osteitis, or keratoma-induced pressure (26–28), with osteitis characterized by demineralization and vascular channel widening (28).

Despite extensive research on the biomechanical effects of horseshoes on equine limbs and hooves, the impact of routine shoeing on radiographic density and morphological adaptations of the distal phalanx remains poorly characterized. The present study aims to investigate associations between dorsal and dorsoabaxial clips and osseous density and structural geometry of the distal phalanx, comparing these associations to unshod hooves. Specifically, we hypothesize that dorsoabaxial clips are associated with (a) localized reductions in radiographic density at the margo solearis beneath clip positions and (b) a narrower in shape distal phalanx configuration, relative to dorsal clips.

## 2 Materials and methods

### 2.1 Selection of radiographs

Radiographic images taken during lameness or pre-purchase examinations at an equine clinic between 2016 and 2023 were retrospectively evaluated. Prior to data collection, the responsible person provided written consent allowing the use of clinical data for research purposes. Radiograph selection was performed using the PACS system with JiveX DICOM software. Inclusion criteria required orthograde Oxspring radiographs of one front foot (either left or right) of Warmblood horses (*n* = 132) or horse-type ponies (*n* = 43; height at the withers: 120–148 cm). Most radiographs had been taken with mildly radiopaque mass in the sulci of the frog. In the following, both horses and ponies are referred to collectively as “equids.” Equids ranging in age from 3 to 28 years were included in the study, with a mean age of 14.1 years. The height at the withers was not available for all equids. Radiographs with artifacts or visible pathologies, whether noted on the image or in the radiology report, were excluded. If radiographs of both front limbs of an individual were suitable, the image deemed superior for measurement was selected.

All radiographic images meeting these criteria were anonymized by assigning each one a unique, randomly generated numeric identifier. This randomization was carried out by computer software employing a pseudo random number algorithm, ensuring equal probability of number assignment. Each radiograph was measured twice, on separate days, by a single investigator blinded to the animal’s identity. Only the numeric codes were accessible during measurements. The average of the two independent measurements was used for further analysis. Equids were grouped into unshod (US), shod with dorsal clip (SC1), and shod with two dorsoabaxial clips (SC2), referred to as groups for the remainder of the paper.

Four age categories were defined based on existing literature (29, 30): 3–6 years (juvenile = JUV), 7–14 years (young adult = YAD), 15–21 years (mature adult = MAD), and >21 years (OLD). A total of 97 male and 78 female equids were included. Due to the low number of stallions (*n* = 2), no distinction based on castration status was made. Based on historical data, the equids were also categorized by use, reflecting increasing workload: leisure equids (LE), dressage equids (DE), and jumping equids (JE). For the remainder of the text, we collectively refer to the parameters age, breed, use, and shoeing type as “horse/pony variables.”

Prior to radiography, horseshoes (if present) had been removed, and the hooves had been trimmed to improve image quality, as is standard practice (31, 32). The person responsible for the equid was asked about the duration of the current shoeing or the barefoot period. Of the 175 equids, 145 had a documented shoeing or barefoot status of at least 1 year prior to the inspection date, while for 30 equids the duration of barefoot or shoeing status was not available. Grouping was done based on the status of the equids presented at the clinic, and the shoeing history was only taken in addition to that, but not as the basis for the grouping of the equids. In order to test the relevance of this additional information, parameters of equids with known 12-month prior history (known history) and equids with unknown 12-month history (unknown history) were compared within each group.

A total of 98 right front and 77 left front Oxspring radiographs (175 equids) were ultimately included. The distribution of equids across groups included 58 in US, 66 in SC1, and 51 SC2. After final selection, each radiograph was assigned a new randomized identification number for analysis.

### 2.2 Image analysis

Using Image J software, 175 selected Oxspring images were analyzed by the first author. This evaluator was blinded to the groups during the measurements and radiographs were analyzed in a random order.

**Figure 1:**
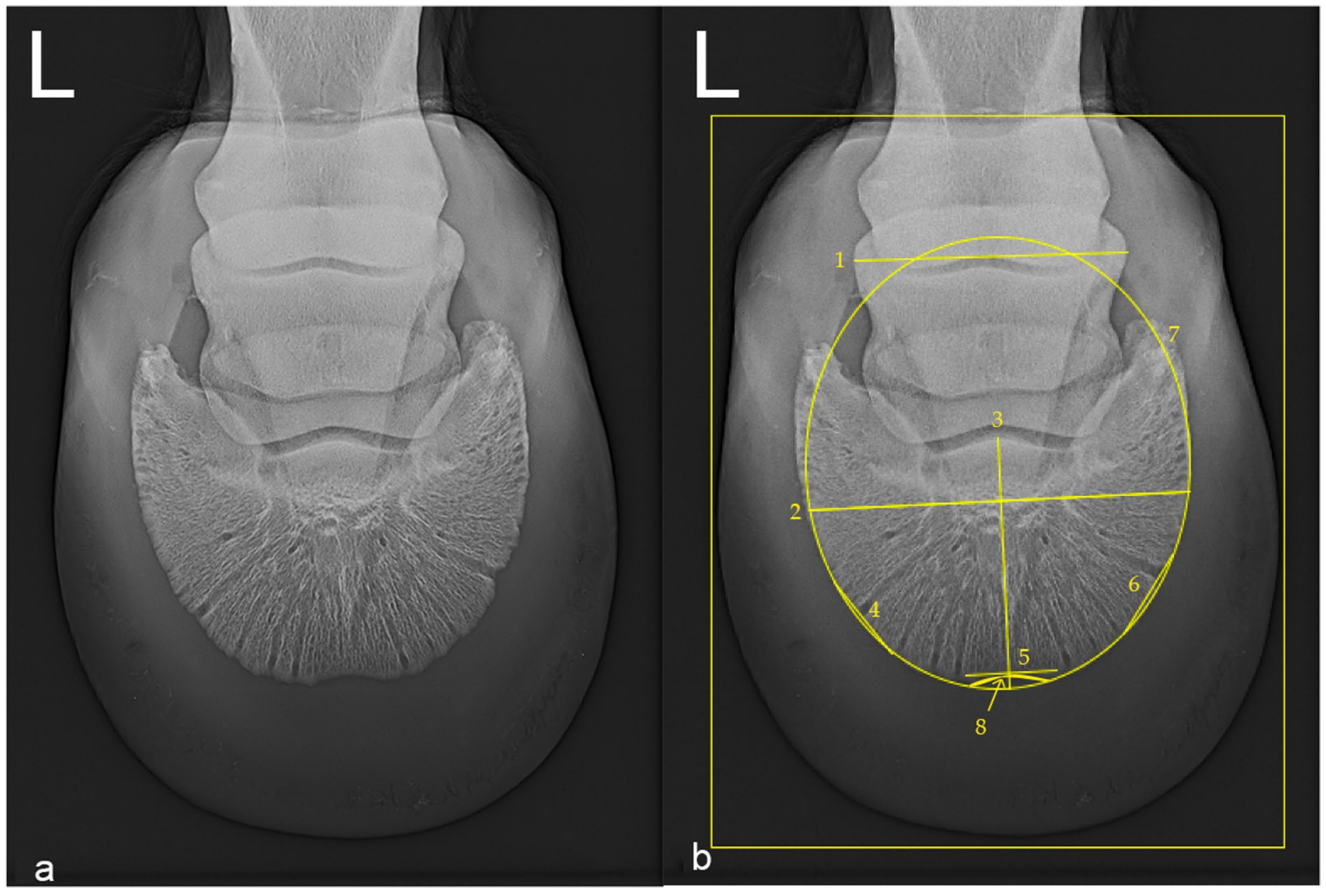
Oxspring radiograph (dorsoproximal-palmarodistal projection) of the left front foot of a 4 year old female leisure Warmblood horse (hoof no. 5), belonging to the unshod (US) group (Diagnostic Imaging, Vetmeduni). Lateral is to the left in both images. **(a)** Native radiograph. **(b)** Radiograph annotated with measurement lines. The width of the radiographic depiction of the middle phalanx (1; 96.3 mm), the relative width (2; 135.62%) and relative length (3; 91.90%) of the distal phalanx were measured using transverse and sagittal reference lines. Lateral (4; 33.33%), dorsal (5; 33.33%), and medial (6; 33.33%) lines were positioned across the distal phalanx to measure gray values. The width to length ratio (WL ratio) is derived from lines two and three, offering a quantitative representation of the shape of the distal phalanx. The shape of the distal phalanx was subjectively classified as elliptical (7). A crena marginis solearis of the distal phalanx is visible (8). Minimum and maximum bone densities as gray values were recorded within the outlined rectangular measurement area.

The relative width of the distal phalanx was measured using a transversal latero-medial line at the widest point of the distal phalanx (Figure 1; Nr.2) and the relative length of the distal phalanx was measured with a sagittal line along the axis of the distal phalanx from the center of the margo solearis to the center of the proximal contour of the extensor process (Figure 1; Nr.3), following previously described methods (33–39). Width to length ratio (WL ratio) was calculated and used to objectively document the shape (Figure 1; Nr.7) of the distal phalanx as near to elliptic (ratio values near or below 1) or round (ratio values 1 or more). All measurements are summarized in Table 1.

**Table 1:** Measurements used in the present study for the analysis of the distal phalanx.

Variable name	Description
Width to length ratio	Calculated ratio of hoof width to length, used to estimate hoof shape.
Distal phalanx width and length	Width and length of the distal phalanx in relation to the width of the middle phalanx in %.
Phalanx media width	Width of the middle phalanx, as measured in the radiograph, in mm.
Dorsal density metrics	Mean, variance, and 10th–90th percentiles of the gray values in the radiograph in the dorsal region.
Medial density metrics	Mean, variance, and 10th–90th percentiles of the gray values in the radiograph in the medial region.
Lateral density metrics	Mean, variance, and 10th–90th percentiles of the gray values in the radiograph in the lateral region.

The presence or absence of a crena marginis solearis was noted (Figure 1; Nr.8). The bone density of the margo solearis was documented at the locations where dorsal and dorsoabaxial clips had been or would have been positioned. Gray values (GV) were obtained along three straight lines as secant lines starting and ending at the margo solearis but intersecting the curvature of the margo solearis (Figure 1; Nr.4, 5, 6), each measuring a third of the relative width of the middle phalanx (Figure 1; Nr.1). Along these lines, GV were collected and referred to as dorsal (D), dorsolateral (L) and dorsomedial (M) gray value series (GVS). The width of the radiographic depiction of the middle phalanx (WRMP) in the images analyzed was used, as not all radiographs had the metal reference marker fully within the collimation. Length measurements of other features of the radiographs were done as percent of the WRMP and only these percentage values were taken forward.

To compare GV between radiographs and to normalize GV in percent resulting in %GV, highest and lowest GV were analyzed within the largest possible rectangle from the hoof. Metal markings, contamination or remnants of shoe nails were excluded. For each GVS the following values were calculated: mean (mn), standard deviation (sd), quantile10 (Q10), quantile25 (Q25), quantile50 (Q50), quantile75 (Q75) and quantile90 (Q90).

### 2.3 Statistical analysis

Statistical analysis of the results was performed using IBM SPSS Statistics, Python, and Microsoft Excel. The data was tested for normality using the Shapiro–Wilk test. When the statistical test showed significant group differences, we conducted a post-hoc analysis to compare pairs of groups. For normally distributed data, we used ANOVA with Tukey HSD as a post-hoc analysis. If the data did not follow a normal distribution, we applied the Kruskal–Wallis test followed by Dunn’s post-hoc test. To assess whether age and group were independent, we conducted an ANOVA. We also used Spearman’s rank correlation (one-tailed) to analyze the relationship between horse/pony variables and %GV.

We conducted an exploratory analysis to assess the stability of WRMP across different groups and use of equids. We evaluated normality using Shapiro–Wilk tests and homogeneity of variances using Levene’s tests. Due to non-parametric distributions in some groups, we used Kruskal-Wallis tests for group comparisons. Additionally, we calculated the Pearson correlation coefficient between the width of the middle phalanx and the WL ratio from the distal phalanx to examine their relationship.

Presence of crena, use of equid, breed, age, WL ratio, shape of the distal phalanx, and %GV (standard deviation, mean, and Q10 to Q90 of dorsal, medial, and lateral %GV) were additionally analyzed separately for warmblood horses and ponies. To evaluate the accuracy of the subjectively assessed distal phalanx shape, we compared the WL ratio between the two shape categories (oval = 0, round = 1). Descriptive statistics were calculated, and a Welch’s t-test was performed to test for significant differences between the groups. A Pearson correlation was computed to assess the relationship between WL ratio and shape. We calculated Cohen’s d to quantify the effect size between US, SC1, and SC2 pairs in relation to %GV.

## 3 Results

Two-way ANOVA revealed a significant main effect of US, SC1 and SC2 on WL ratio (*F* = 13.21, *p* < 0.001), while the fact, that known or unknown history showed no significant main effect (*F* = 0.78, *p* = 0.379) or interaction with US, SC1 and SC2 (*F* = 1.02, *p* = 0.413). Descriptive statistics and Dunn’s post-hoc tests with Bonferroni correction yielded consistent results across equids with known duration of barefoot trimming or shoeing and those without such historical data. This consistency was observed in both the magnitude of effects and patterns of statistical significance. Comparison of mean and standard deviation of WL ratio of distal phalanx among group unshod (US), shod with one dorsal clip (SC1), and shod with two dorsoabaxial clips (SC2), stratified by the presence or absence of a known shoeing or trimming history over the previous 12 months, showed no significant effect of shoeing history on the WL ratio within any group. For unknown: US (mean = 1.35, min = 1.17, max = 1.45), SC1 (mean = 1.39, min = 1.25, max = 1.57), SC2 (mean = 1.30, min = 0.7, max = 1.55). For known history: US (mean = 1.37, min = 0.8, max = 1.82), SC1 (mean = 1.41, min = 0.9, max = 1.75), SC2 (mean = 1.32, min = 0.86, max = 1.66). Significant differences were found between SC1 and SC2 in both subgroups (unknown history: Test Statistic = 3.752; *p* = 0.00093, known history: Test Statistic = 3.928; *p* = 0.00074).

There were no significant differences between radiographs of left and right distal phalanges in any of the variables examined, therefore left and right forelimb results are presented together. No significant difference in WL ratio was observed between the groups of Warmbloods and ponies (H-statistic = 2,78, *p* = 0.25).

We assessed normality (Shapiro–Wilk) and homogeneity of variances (Levene’s) for WRMP (mean = 68.01 mm, sd = 5.05 mm) across groups and use of equid. Due to violations of assumptions, we employed Kruskal-Wallis tests. Results showed significant differences in WRMP for groups (*p* = 0.0057) and use of equid (*p* = 0.0331). The Pearson correlation between WRMP and WL ratio was negligible (*r* = 0.008), indicating their independence.

The WL ratio differed significantly between oval and round hooves, (oval: mean 1.301 sd, round: mean 1.396 sd; t = −3.935, *p* < 0.001).

**Figure 2:**
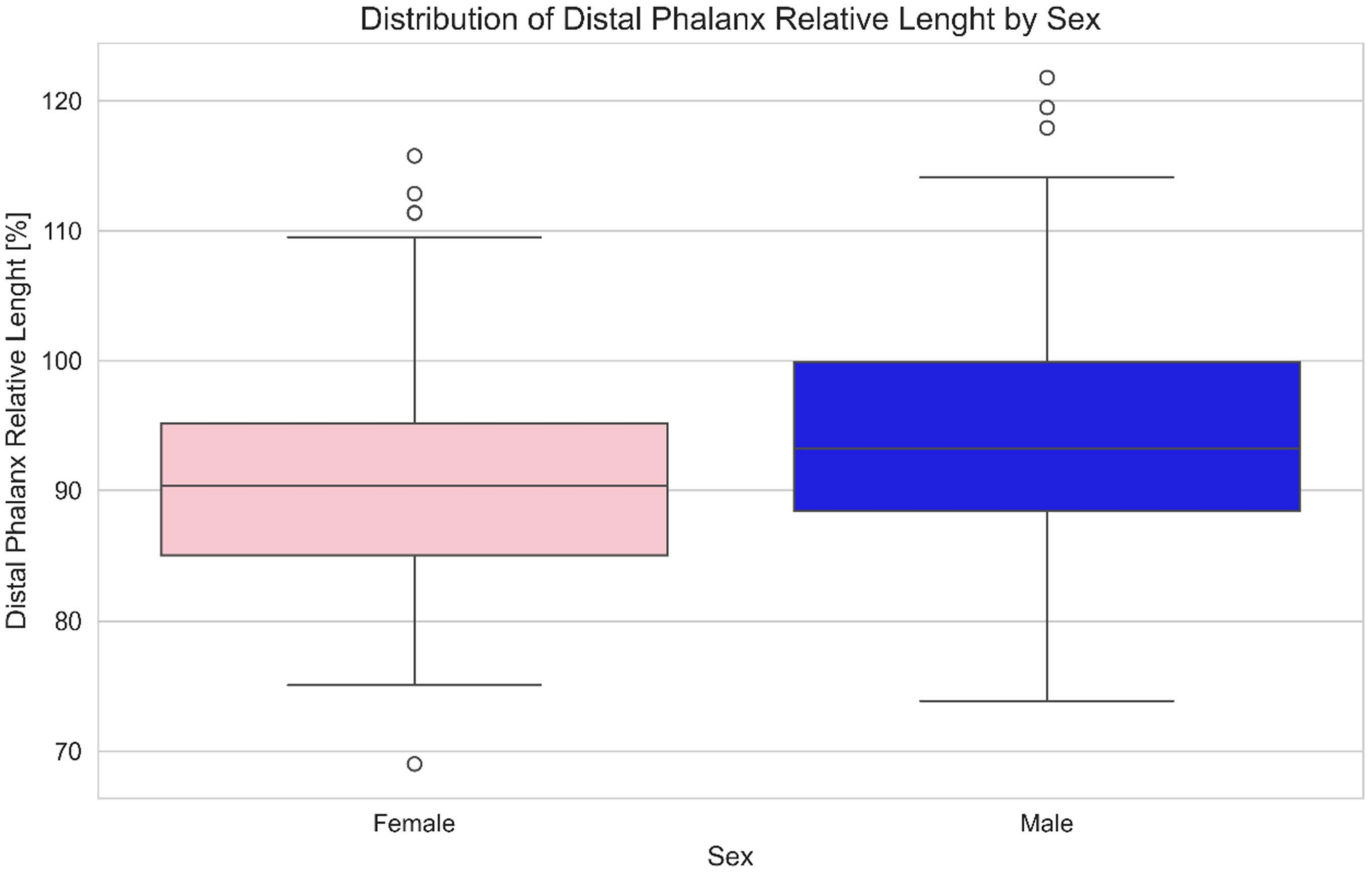
Distribution of relative length of the distal phalanx length (%) by sex in equids. This boxplot based comparison highlights potential differences in relative distal phalanx length between male and female subjects. Each box represents the interquartile range (IQR), with the median indicated by a central line and whiskers extending to the full range of data (the circles O represent possible outliers). Pink boxes represent females and blue boxes represent males, offering a clear visual distinction of central tendency, dispersion, and outliers for each sex. One extreme outlier in the male group was ignored for the clarity of the visualization.

**Figure 3:**
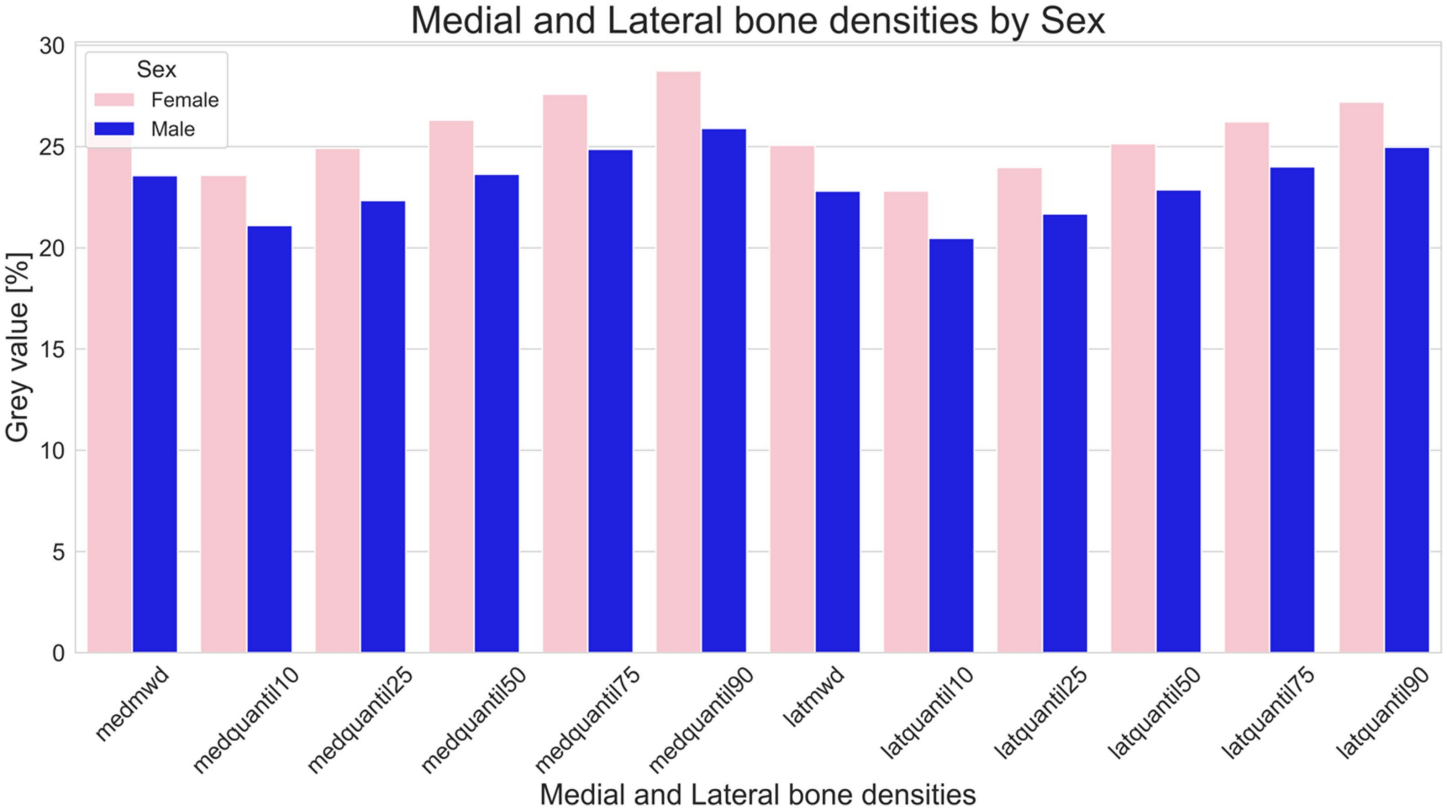
Comparison of bone density in male (blue) and female (pink) equids. This figure illustrates variations in as median gray value percentages (indicative of bone density), and the 10th, 25th, 50th, 75th, and 90th Quantile of the medial and lateral distal phalanx density of the distal phalanx. Bars represent the sum of the medial and lateral gray value series (GVS). Sums of GVS were significantly higher in female than in male equids (medial *p* = 0.007; lateral *p* = 0.016).

A statistical difference between male (predominantly castrated) and female equids was found for relative length of the distal phalanx (means male 91.30%, female 95.43%. T-statistic 2.1285; *p* = 0.0347) and for medial and lateral GV% distributions (medial means male = 0.2356, female = 0.26228. T-statistic 2.7002; *p* = 0.007; lateral means male = 0.2279, female = 0.2506. T-statistic 2.4073; *p* = 0.017) (Figures 2, 3).

Age and groups US, SC1 and SC2 contribute to the variance in WL ratio independently with a two-way ANOVA (*F*-value = 18.23; *p* < 0.00001 for age and *F*-value = 5.56; *p* = 0.00456 for the groups, respectively). No significant interaction effect was found between the age and the groups (*F*-value = 0.94; *p* = 0.39). In equids, higher GV% were seen medial (SC1 = 0.01806, SC2 = 0.01938) and lateral (SC1 = 0.0174, SC2 = 0.0179) in SC2 compared to SC1, but this was not significant (Medial Mann–Whitney U = 1,478.6; *p* = 0.257. Lateral Mann–Whitney U = 1,813.0; *p* = 0.4765). Between SC1 and SC2 differences were found in the medial (SC1 mean = 0.235, sd = 0.056, min = 0.137, max = 0.394; SC2 mean = 0.235, sd = 0.068, min = 0.151, max = 0.422) and lateral (SC1 mean = 0.233, sd = 0.054, min = 0.157, max = 0.381; SC2 mean = 0.242, sd = 0.068, min = 0.138, max = 0.421) GV%. In US equids the medial GV% are higher than in SC1, although not strong enough to be significant (US mean = 0.257, sd = 0.071; SC1 mean = 0.235, sd = 0.0558; SC2 mean = 0.25298, sd = 0.0684).

Table 2 shows the radiographic dimension of the middle phalanx length, the relative distal phalanx width and length and WL ratio. No significant differences were found between the relative width of the distal phalanx of SC1 and SC2 (*H* = 3.27, *p* = 0.195). However, significant differences were found in the relative length of the distal phalanx between groups (*H* = 9.33, *p* = 0.0094).

**Table 2:** Width of the radiographic depiction of the middle phalanx (WRMP in mm), relative width of the distal phalanx (% of WRMP), relative length of the distal phalanx (% of WRMP), and the width to length ratio of the distal phalanx among three groups of equids: unshod (US), shod with one dorsal clip (SC1), and shod with two dorsoabaxial clips (SC2).

	WRMP [mm]			Relative width of distal phalanx			Relative length of distal phalanx			Width to length ratio		
Group	Mean	Min	Max	Mean	Min	Max	Mean	Min	Max	Mean	Min	Max
US	67.81	55.35	78.75	126%	91%	174%	92%^a^	75%	122%	1.37	0.80	1.82
SC1	68.31	60.75	80.55	128%	98%	163%	92%^a^	69%	118%	1.40^b^	0.89	1.75
SC2	66.59	53.80	79.80	126%	83%	159%	98%^a^	79%	180%	1.31^b^	0.70	1.66

Shaded cells and identical superscript letters indicate significant differences between values. ^a^*H* = 9.33, *p* = 0.0094; ^b^*H* = 13.01, *p* = 0.0015.

Group US exhibited an average WL ratio of 1.37 (median = 1.38, min = 0.8, max = 1.82). Group SC1 had a slightly higher mean ratio of 1.40 (median = 1.41, min = 0.9, max = 1.75). In contrast, group SC2 demonstrated a significantly lower mean ratio of 1.31 (median = 1.3, min = 0.7, max = 1.66). Groups were significantly different overall (*H* (2) = 13.01, *p* = 0.0015), with group SC2 showing a significantly lower WL ratio compared to SC1 (*p* = 0.00093), whereas no significant differences were documented comparing US and SC2 (*p* = 0.159) and comparing US and SC1 (*p* = 0.284).

**Figure 4:**
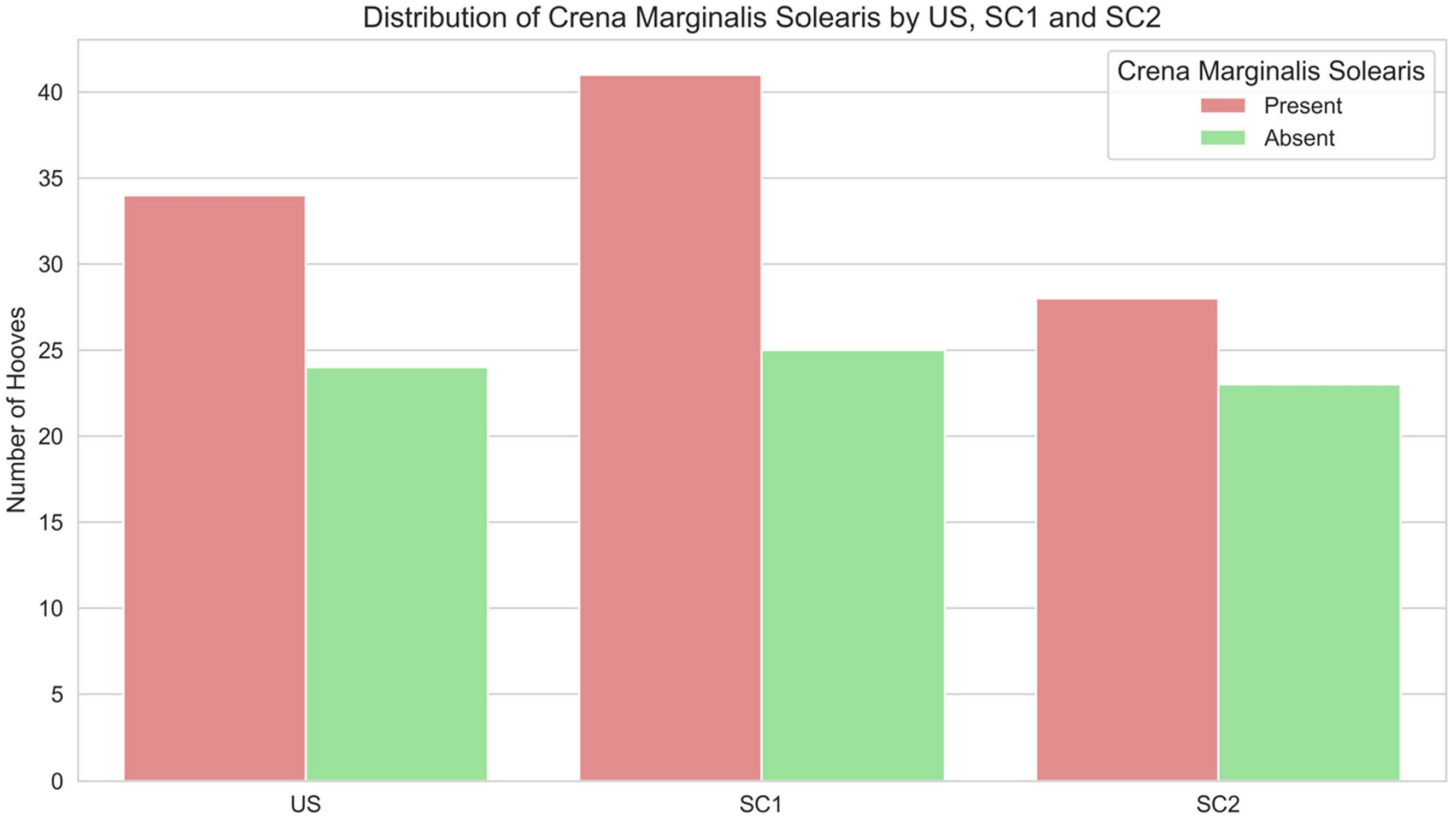
Distribution of presence (red bars) and absence (green bars) of a *crena marginalis solearis* in distal phalanges of equids of unshod hooves (US), and of hooves shod with a single dorsal clip (SC1), or with two dorsoabaxial clips (SC2).

The presence of a crena marginalis was independently associated with greater distal phalanx relative width and a higher WL ratio. Equids with a crena exhibited a significantly higher distal phalanx relative width (mean difference = 0.049, 95% CI [0.028, 0.071], *p* = 0.024), independent of group US, SC1, SC2. The WL ratio was significantly higher in individuals with a crena (mean difference = 0.082, 95% CI [0.059, 0.106], *p* < 0.001), with group US, SC1 and SC2 showing no independent effect (χ^2^ = 0.62, *p* = 0.73). There was no association between the presence of a crena and group US, SC1 and SC2 (Figure 4). Breed differences remained evident: warmbloods were overrepresented among equids with a crena, whereas ponies were more likely to have no crena (χ^2^ = 9.29, *p* = 0.0096).

**Figure 5:**
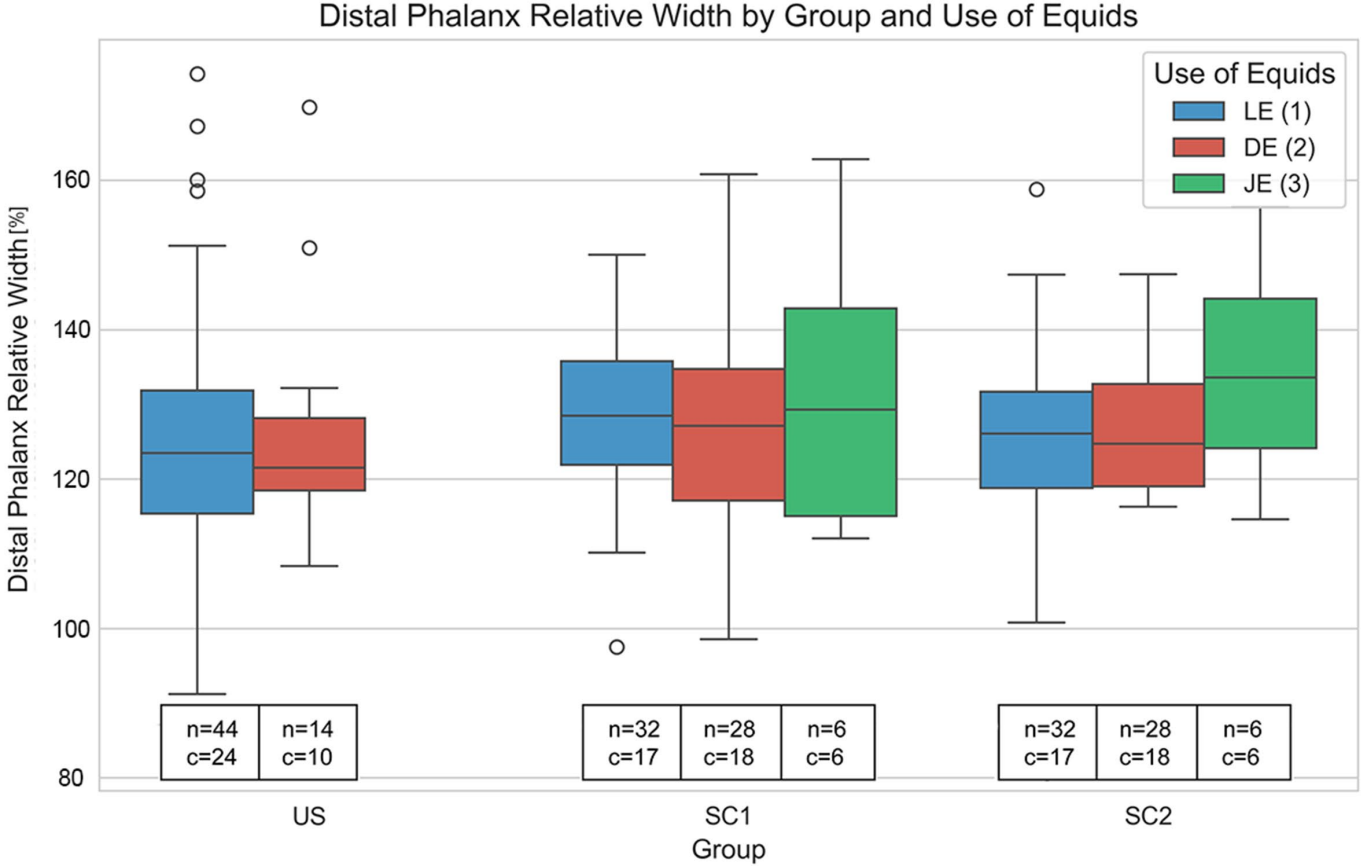
Variation in relative distal phalanx width (%) across unshod (US), single dorsal clip (SC1), and two dorsoabaxial clips (SC2) groups, further subdivided by use of equids (LE: leisure, DE: dressage, JE: jumping). Each boxplot shows the distribution of relative distal phalanx widths within each category, including median values, interquartile range (IQR), and outliers (O). Text annotations indicate sample sizes (n) and the presence of crena marginalis solearis (c). Notably, individuals with a crena show significantly greater distal phalanx relative width (*p* = 0.001), independent of US, SC1 and SC2 or use, with warmbloods overrepresented in the group with a crena marginalis solearis. These results highlight the crena as an important determinant of distal phalanx morphology, with potential breed specific effects.

These findings indicate that the crena marginalis is a key factor associated with the shape of the distal phalanx, particularly its mediolateral dimension, and that its distribution varies significantly across warmbloods and ponies (see Figure 5).

**Figure 6:**
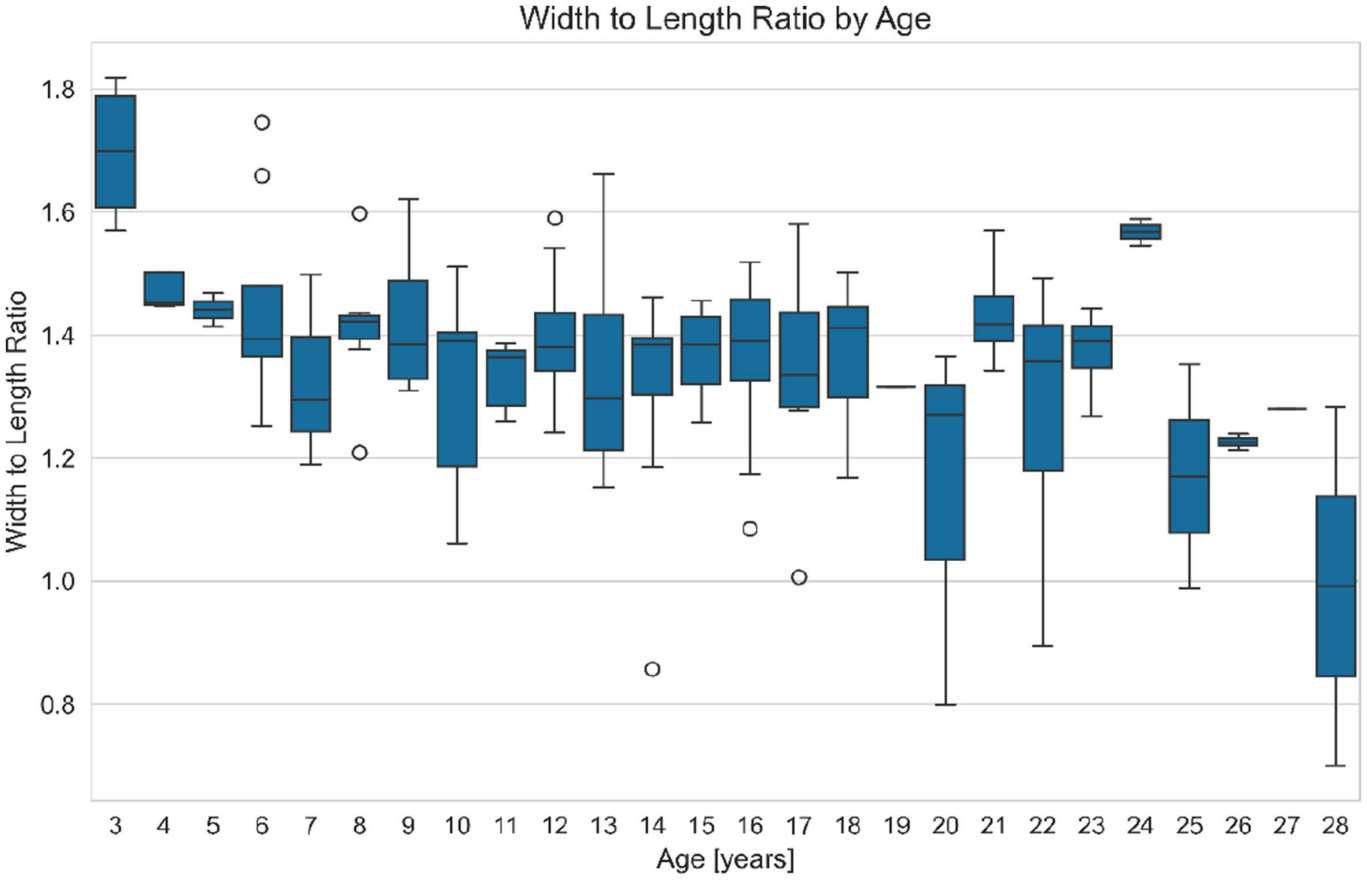
Variation of the distal phalanx width to length ratio (WL ratio, a dimensionless value) with age in equids. This figure presents boxplots of the WL ratio grouped by age, illustrating how hoof proportions change as equids grow older. Each boxplot shows the IQR (Interquartile Range), outliers (O), the range between the first quartile (25th percentile) and the third quartile (75th percentile) of a dataset, with the median value indicated. The results suggest that older equids tend to have a lower WL ratio, and thus a relatively narrower distal phalanx, compared to younger equids.

Comparing all age groups WL ratio and age differ significantly (JUV = 1.47197, YAD = 1.3776, MAD = 1.3785, OLD = 1.33446. Kruskal H-value = 19.0998; *p* = 0.0003), and WL ratio of the youngest group JUV (median = 1.47 min = 1.25, max = 1.82) differs significantly from group YAD (median = 1.38, min = 0.86, max = 1.66. Mann–Whitney U = 1,221.0; *p* = 0.00011), from group MAD (median = 1.37, min = 0.80, max = 1.58. Mann–Whitney U = 835.0; *p* = 0.00038) and from group OLD (median: 1.36, min = 0.70, max = 1.59. Mann–Whitney U = 361.0; *p* = 0.00112) (Figure 6).

**Figure 7:**
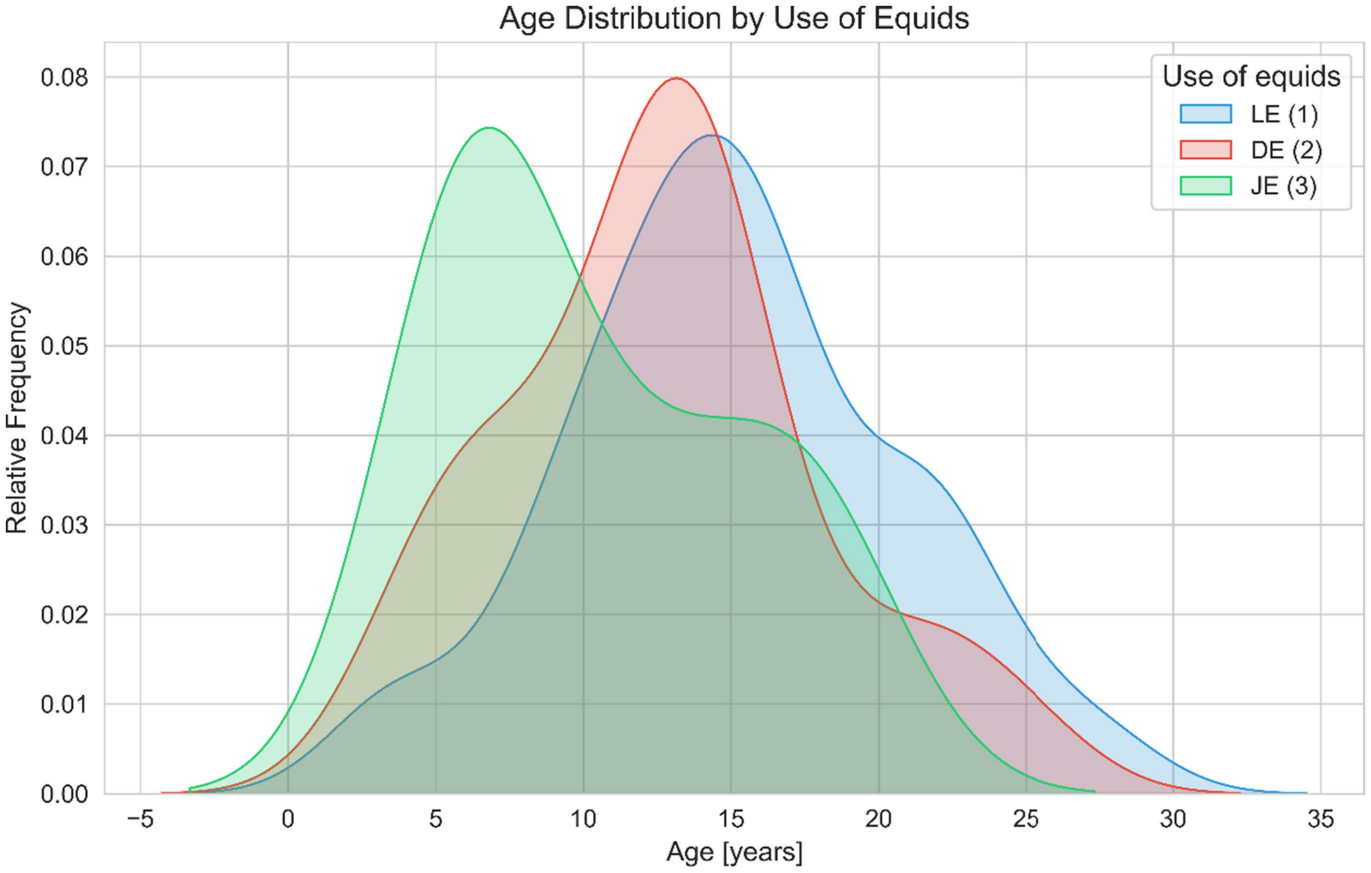
Age distribution across different uses of equids (leisure LE, dressage DE, and jumping JE). This figure depicts Kernel Density Estimation (KDE) plots of age for LE (blue), DE (red), JE (green). The shaded areas represent the relative frequency of equids within specific age ranges, facilitating visual comparison of age-related demographics across different categories of use. The data suggest that leisure equids tend to be older than those used for jumping or dressage.

The age distribution for LE peaks between approximately 15 and 20 years, DE shows a peak in equid use between 10 and 15 years of age. In contrast, the JE exhibits its highest density of equid use at the age of 5–10 years. Leisure equids are older than jumping or dressage equids (Figure 7).

No significant differences in the relative distal phalanx width were found for the use of equids. The mean relative widths were 1.2626 for LE, 1.2688 for DE, and 1.3311 for JE, with the Kruskal–Wallis test showing a non-significant result (*H* = 1.0224, *p* = 0.5998) (Figure 5).

The single tailed correlations of the radiographs of the distal phalanx of warmblood horses and the single tailed correlations of the radiographs of the distal phalanx of ponies are shown in Tables 3, 4.

**Table 3:** Significant correlations of gray values in percentage (based on the gray value range of each individual radiograph) of the dorsal, medial, and lateral lines of the distal phalanx in Warmblood horses (*N* = 132).

Warmblood		Variable name	US SC1 SC2	Use of horse	Age of horse	Width to length ratio
			PCC	PCC	PCC	PCC
Horse variables		Age of horse	−0.098	−0.338^A^		
		Shape of distal phalanx	−0.204^b^	−0.073	−0.001	
		Width to length ratio	−0.093	−0.123	−0.208^C^	
Gray values in percentage per image	Dorsal	Standard deviation	−0.189^d^	−0.005	−0.173^e^	−0.124
	Medial	Mean	−0.023	−0.099	−0.084	−0.166^f^
		Standard deviation	−0.041	−0.082	−0.044	−0.224^G^
		Quantile25	−0.038	−0.097	−0.084	−0.157^h^
		Quantile50	−0.022	−0.100	−0.086	−0.173^i^
		Quantile75	−0.012	−0.103	−0.080	−0.176^j^
		Quantile90	−0.016	−0.102	−0.086	−0.178^k^
	Lateral	Standard deviation	−0.004	−0.054	−0.152^l^	−0.094

Shaded cells and superscript letters denote significant correlations, while capital letters indicate highly significant correlations. Group definitions: Unshod (US), shod with one dorsal clip (SC1), and shod with two dorsoabaxial clips (SC2); use categories: leisure, dressage, jumping; age in years; width to length ratio of the distal phalanx; Mean, standard deviation (SD), Quantile25, Quantile50, Quantile75, and Quantile90 are given in percentages. PCC denotes the Pearson correlation coefficient.

A: *p* = 0.0001; b: *p* = 0.010; C: *p* = 0.009; d: *p* = 0.015; e: *p* = 0.024; f: *p* = 0.029; G: *p* = 0.005; h: *p* = 0.037; i: *p* = 0.024; j: *p* = 0.022; k: *p* = 0.021; l: *p* = 0.041.

**Table 4:** Significant correlations of gray values in percentage (based on the gray value range of each individual radiograph) of the dorsal and medial lines of the distal phalanx in ponies (*N* = 43).

Pony		Variable name	US SC1 SC2	Use of pony	Age of pony	Width to length ratio
			PCC	PCC	PCC	PCC
Pony variable		Age of pony	−0.385^A^	−0.058		
		Width to length ratio	−0.305^b^	−0.131	−0.534^C^	
Gray values in percentage per image	Dorsal	Standard deviation	−0.231	−0.069	−0.146	−0.320^d^
	Medial	Quantile10	−0.070	−0.258^e^	−0.165	−0.113

Gray cells and superscript letters denote significant correlations, with capital letters indicating high significance (*p* < 0.01). Group definitions: Unshod (US), shod with one dorsal clip (SC1), and shod with two dorsoabaxial clips (SC2); use categories: leisure pony, dressage pony, jumping pony; age in years; width to length ratio of the distal phalanx; Mean, standard deviation (SD), and Quantile10 are presented in percentages. PCC denotes the Pearson correlation coefficient.

A: *p* = 0.005; b: *p* = 0.023; C: *p* = 0.0001; d: *p* = 0.018; e: *p* = 0.047.

## 4 Discussion

The present retrospective study investigated the association of dorsoabaxial clips with the radiographic shape of the equine distal phalanx of forelimbs and documented a significantly longer distal phalanx relative length in hooves shod with dorsoabaxial clips compared to hooves shod with one dorsal clip, leading to a lower width to length ratio. The hypothesis that the distal phalanx of equids shod with two dorsoabaxial clips differs significantly in shape compared to equids shod with one dorsal clip is supported, specifically due to increased relative length rather than a narrower width. The hypothesis regarding the reduced density at the clip sites is not supported. To the authors’ knowledge, this is the first study documenting the association of dorsoabaxial clips with the shape of the distal phalanx.

The present study is retrospective and the Oxspring radiographs of the forelimbs selected were originally taken for clinic necessities. The advantage of a retrospective study is that many radiographs of the distal phalanx were available, and the goal of a relatively simple and cost effective method (40) to show the effects of horseshoes with dorsoabaxial clips was achieved. In a previous study an even larger number of lateromedial radiographs of the distal phalanx of forelimbs was used to measure the concavity of the parietal solar surface of the distal phalanx of equids, which was negatively correlated with age (41). The sample size in the present study did not reach the high number reported in the previous study (41), where 175 Oxspring radiographs of equids taken during lameness or prepurchase examinations were analyzed. Similar to the above report on the solear surface of distal phalanges (41), only digital radiographs were used in the present study, as they provide consistent and high quality images that facilitate detailed evaluation of bone structures and gray value analysis for assessing bone density.

In horses, middle phalanx dimensions, distal phalanx dimension and navicular bone dimensions show strong correlations and size ratios were similar in ponies, saddle horses and harness horses (42). Based on this relationship, middle phalanx width measured on digital Oxspring radiographs was used as a reference in the present study, documenting a relationship between dorsoabaxial clips and bone density changes and distal phalanx shape, one of the main areas of interest in the distal phalanges (43). Radiological bone size measurements are usually done in standard radiographs to investigate distal phalanx morphology, for example proximodistal shortening of the distal phalanx in laminitis (43). Similar to our study, relative measurements in radiographs are frequently used, in order to overcome the difference between true length measurements and measurements of the same structures in the radiograph, where magnification of an unknown magnitude is present (44).

Oxspring radiographs highlight the solear margin of the distal phalanx, where bone remodeling or lysis can be seen (45), this is also the anatomical feature that the present study is focusing on. Either left or right Oxspring radiographs of any individual equid were used in the present study for further processing, creating a data set where each individual equid was represented only once. As demonstrated in a previous study, which found no radiographic differences between left and right front distal phalanges (46), we also observed no significant differences in our analysis. Therefore, when radiographs of both front distal phalanges were available, the one more suitable for further processing was selected. The selection criteria for artifact free radiographs in this study align with those of a previous investigation on equine distal phalanges that utilized ImageJ to document arterial channel changes (47). Consistent with previous studies (48, 49), evaluation of radiographs in the present study relies on subjective assessment. The present study shows that trained observers can reliably distinguish between narrow and wide distal phalanges from Oxspring radiographs without precise measurements. The alignment between expert visual assessment and quantitative measurements highlights the importance of experienced radiographic interpretation. The ability to make accurate assessments without time consuming measurements is thus invaluable in day to day veterinary work (50), even though subjective evaluations are about to be overtaken by the use of artificial intelligence (51).

After selection of all radiographs of the present study, radiographs were processed in random order, thus, for all intents and purposes, the evaluator was blinded to patient data, history, examination findings, and radiography report. This randomization process was implemented to minimize bias, a method also employed in a previous study assessing measurement variability in osteophytosis scoring systems (52). To address potential size differences in digital radiographs (52), measurements were normalized to the width of the middle phalanx, as not all radiographs had metal markers within the collimation. Recording hoof dimensions before or exact distances to the plate and the generator during radiographic examination was not done, as these measurements were not compatible with routine clinical practice in live equids or the retrospective nature of the study. Disadvantages of evaluating digital radiographs over 3D depictions such as CT, include magnification, summation, and superimposition of bone and soft tissue (53). Similar to our study, relative measurements in radiographs are frequently used, in order to overcome the difference between true length measurements and measurements of the same structures in the radiograph, where magnification of an unknown magnitude is present (44). To mitigate potential confounding effects arising from variations in radiographic technique and patient positioning, the width of the distal phalanx and all other length measurements in the present study were normalized to the width of the middle phalanx, as internal reference markers were absent. This normalization strategy aligns with the methodology described by a previous study (54), which advocated for the use of vertebral body lengths as a normalizing factor for tracheal height measurements in equine radiographs. It was posited that ratios utilizing adjacent osseous landmarks facilitate more robust intersubject comparisons. The normalization to middle phalanx width in the present study was intended to minimize the impact of inconsistent magnification, mirroring normalization strategies employed in prior studies that assessed skeletal dimensions. A recent study acquired lateromedial radiographs of the front feet of unsedated draft horses and calculated the ratio of dorsal hoof wall thickness to the distal phalanx length, also highlights the importance of proportional measurements in radiographic assessment of the equine digit, demonstrating the utility of the ratio of dorsal hoof wall thickness to the length of the distal phalanx in evaluating dorsal hoof wall thickness (55). Similar ratiometric approaches have been used in other species. For example, an other non equine study used vertebral measurements to normalize cardiac dimensions in radiographic studies of mice (56).

As in previous studies, gray values were used to evaluate bone density, aiming to assess the precision and accuracy of bone mineral density measurements from digitized radiographs (57). To ensure comparability between radiographs, we used relative density ranges (0–100%) within each image, excluding nails and other metal dense structures from the gray value range. This approach eliminated the need for aluminum as a contrast agent, as used in some previous studies (58, 59) and allowed for comparison of radiographs processed with different algorithms, which were repeatedly adapted to improve image quality by the radiographers over the time from which the radiographs were sourced.

In the present study, equids only from the age of 3 years onwards were included, as the majority of growth plates are closed by this time (60). This approach aligns with a previous study on navicular disease, which also excluded young equids (61). As demonstrated in this previous study, certain navicular bone shapes are predisposed to developing navicular disease, highlighting the importance of bone shape in equine health (61). Similarly, our study focuses on the distal phalanx, raising the possibility that its shape also plays a role in disease development.

For the aim of the present study, it was imperative that equids were at an age when shoeing would have been a possibility or had been carried out. The present study focused on equids aged 3 years and older, a pivotal age when equids conventionally initiate both athletic training and hoof management through routine shoeing (62, 63). Similar to the population investigated in the present study, the study population of a previous study using radiographic analysis in equids (29, 30) was identified as juvenile, young adults, mature adults and old equids. Regarding the use of the equids, the study population of the present study was grouped as: leisure, jumping or dressage, representing main types of equestrian disciplines, as seen in previous research how management and training practices influence back mechanics and flexibility of equids (64). Dressage equids showed a 33% larger mean degree of motion at L3 than leisure equids and 19% more than show jumpers, highlighting significant differences in back flexibility among these equestrian group (64). In the present study is seen, that show jumpers and dressage equids were younger than leisure equids. This is represented in other large studies of the equine population (65, 66). Performance of dressage and jumping equids increased linearly until age 10, then plateaued in both disciplines (65) with another study highlighting that jumping equids reach peak performance at younger ages compared to dressage equids (66). The present findings on age distribution among show jumpers, dressage equids, and leisure equids align with previous research (64, 65). Grouping into dressage, jumping and leisure equids in the present study allowed for exploration of differences in athletic management practices between competition equids and leisure equids. Additionally, no differences in distal phalanx morphology, within the different uses of equids are seen. This lack of variation contrasts with the different biomechanical demands of various disciplines reported in a previous study (50), suggesting that distal phalanx morphology may be less influenced by use than previously thought.

To understand the more pronounced effect of dorsoabaxial clips on distal phalanx shape and density in the present study compared to a single dorsal clip, it’s essential to consider equine hoof mechanism. Barefoot trimmers often argue against shoeing due to its interference with hoof biomechanics, as heel widening is limited even with horseshoes without clips (15). Dorsoabaxial clips exert greater pressure on the hoof and distal phalanx (14). During the stance phase of locomotion, the hoof undergoes significant deformation. The distal phalanx’s downward force causes the suspensory apparatus to pull the corresponding hoof wall section downwards up to 1.5 mm, most pronounced at the coronary band and decreasing distally (67), where clips are located. Based on the results of the present study, dorsoabaxial clips, typically positioned between the first and second nail, affect the distal phalanx despite one study suggesting less impact in this area compared to clips palmar to the second nail (2). The hoof narrows proximally and expands distally palmar to the widest transverse distance of the coronet and the distal margin of the hoof wall, resulting in an outward movement of the distal parts of the hoof wall (67). Simultaneously, the distal phalanx pushes the sole and frog into contact with the ground, causing the solar arch to flatten and the weight bearing edge to widen behind the broadest section of the hoof (67). The observed changes in bone density near the clip areas and the shape changes of the distal phalanx in the present study suggest potential biomechanical effects of this shoeing method. These findings are further corroborated when comparing the distal phalanges of shod and unshod equids. The influence of shoeing on distal phalanx shape is more pronounced between different shoeing types rather than between shod and unshod hooves. The shape differences observed in equids shod with two dorsoabaxial clips are likely attributable to the position of the two clips rather than the presence of a shoe itself. Furthermore, distal phalanges from equids shod with one dorsal clip exhibited greater similarity to distal phalanges from unshod equids than equids shod with two dorsoabaxial clips. A single dorsal clip is preferable because it does not interfere with the natural expansion of the heels, allowing for more normal hoof use (68, 69). This observation supports the hypothesis that the position of the clip(s) of horseshoes plays a more significant role in distal phalanx morphology than the presence of shoes. These findings are explained by previous research using finite element analysis, which demonstrated that higher pressure acts on the hoof in the area of dorsoabaxial clips compared to a dorsal clip (14), which may exert force on the distal phalanx during the stance phase.

The observed changes in distal phalanx density and shape can be explained by bone remodeling in response to altered load distribution (70). At birth, specific trabecular bone parameters of bones of Warmblood versus Shetland pony foals show that genetic and intrauterine developments are relevant to prepare them for the specific mechanical loading of getting up and moving very quickly after birth (71). With loading, bone is continuously adapted to its function based on Wolff’s law from the late 19th century. Bone adaptation continues to be a significant area of study in veterinary research (72), particularly in horses, the species most closely associated with locomotion. The changes in distal phalanx density and shape observed in the present study can be explained by bone remodeling in response to altered load distribution (70). Functional adaptation of the osseous tissues of the navicular bone of horses has also been described (73). Bone is a dynamic tissue comprising specialized cells such as osteoclasts and osteoblasts, which engage in complex interactions within basic multicellular units to drive the bone remodeling process (74). The balance between bone formation and resorption can be disturbed by various factors such as increasing age, estrogen deficiency, calcitriol deficiency, glucocorticoids (75–77), and also increased pressure on the bone, as observed in our study. Additionally, increased pressure on bone leads to bone remodeling (74). This phenomenon is not limited to equines but is also observed in human medicine, for example when the phalanges in fingers change after prolonged wearing of tight rings creating localized pressure on the bone even though the soft tissues remain less changed (78). In cases of such chronic ring erosion injuries, persistent circumferential pressure leads to notable bone modifications of structure and density, which can be detected through radiographic imaging (78).

The crena marginalis solearis is an anatomical feature at the dorsal border of the solar margin of the distal phalanx, representing a normal anatomical variant with individual and breed-related differences (79–81). Consistent with previous studies, we found no association between the presence of a crena and shoeing, especially the use of a dorsal clip (38, 82, 83), indicating that the presence of a crena is no contraindication for dorsal clips.

Our results highlight a significant relationship between the presence of a crena and a wide relative distal phalanx with and high width to length ratio, key parameters of distal phalanx shape variation. This suggests the crena may indicate fundamental anatomical differences, possibly reflecting breed specific or adaptive traits (84). The higher prevalence of the crena in warmbloods compared to ponies aligns with documented differences in the morphology of the distal phalanx (80). While the underlying causes, genetic, developmental, or biomechanical, remain unclear, absence or presence of the crena should be considered in morphometric and clinical distal phalanx assessments. Given its influence on principal shape dimensions, we suggest including the presence of the crena marginalis as a covariate in future studies of equine hoof and distal phalanx morphology.

From the results of the present study no conclusions should be drawn about the effects of clips on the distal phalanx of the hindlimbs. The hind hoof is usually narrower and has a different morphology (85) and is subject to different ground reaction forces, with forelimbs experiencing greater vertical and earlier horizontal loads than hindlimbs (86). These differences likely result in varying degrees of hoof deformation during stance, with less widening in the hindlimbs (87). Consequently, the hindlimbs may never reach the counterpressure from abaxial clips that are observed in the front limbs.

While the present study provides the first evidence of distal phalanx shape deformation associated with dorsoabaxial clips, limitations must be considered. Additional information on body mass, foot shape and quality, cold or hot shoeing, shoe specifications beyond the clips, and work history was not available, even though these factors may play an important role; therefore, they should be considered for future studies. Although there were no statistical differences in the density of the distal phalanx in the area of the dorsoabaxial clips, a trend was observed suggesting higher density in this region compared to equids shod with a dorsal clip. We hypothesize that the pressure exerted by the clips leads to bone remodeling and subsequently to an increase in bone density. This hypothesis is supported by previous research demonstrating that continuously applied pressure and cyclic pressure, such as that exerted by clips, can lead to bone resorption through osteoclastogenesis and osteoclast activity (88, 89) and adjacent bone density increases (90).

The present study did not measure the pressure exerted by dorsoabaxial or dorsal clips on the hoof wall and subsequently on the distal phalanx. However, considering the results of a finite element hoof model from a previous study, it has been shown that horseshoes with two dorsoabaxial clips can exert pressure values of 5.36–6.89 N/mm^2^ onto the medial and lateral hoof wall where dorsoabaxial clips are located (14). This study tested four different types of horseshoes on a hoof model with a total loading of 3,000 N onto the inside of the hoof wall (14). Dorsoabaxial clips were found to reduce outward displacements of the weight bearing rim of the hoof wall in their position by 25–50 μm compared to 100–275 μm wall displacement observed with horseshoes having no clips or one dorsal clip (14). A previous study confirmed that local pressure induced bone resorption results from mechanical forces like cyclic or continuous pressure, leading to the breakdown of bone (88). Continuous compressive pressure from 0.05 N/mm^2^ to 0.2 N/mm^2^ significantly inhibited the differentiation of mouse osteoblast like cells, possibly through prostaglandin E2 mediation, leading to an impact on bone formation and resorption (88). Pressure leading to bone resorption even without direct contact was shown as bone resorption was triggered through cyclically applied fluid pressure in alive rats (89). The tibiae of 50 rats went through maximum 0.1 N/mm^2^ and plateau levels of 0.05 N/mm^2^ intermittent fluid pressure and bone volume, bone volume fraction, and bone mineral density were measured using Microcomputer tomography (89). A correlation between maximum fluid pressure and fluid pressure plateau and bone volume fraction was seen. Bone resorption was measured as effect of the local fluid pressure (89). As the previous two studies have shown, relatively low pressures between 0.05 N/mm^2^ and 0.2 N/mm^2^ lead to bone density changes.

Findings from finite element analyses help explain the increased bone density in warmblood horses shod with dorsoabaxial clips and the distal phalanx shape changes observed in the present study. Hoof conformation is clearly related to the forces applied to the equine foot (61). It is important to emphasize that a relatively longer distal phalanx with an unchanged width, resulting in a lower width to length ratio, could potentially affect hoof biomechanics and stability, even when subjected to the same forces (91). In Thoroughbred racehorses hoof size, shape and balance are related to musculoskeletal injuries as suspensory apparatus failure and fractures of the third metacarpal bone. Musculoskeletal injuries were 0.62 times lower for a 5 mm increase in ground surface of the hoof (34). Thus, morphological distal phalanx change could potentially predispose equids to other conditions or injuries, a consideration that warrants further investigation.

The consistency of results between equids with known and unknown shoeing and barefoot history durations may indicate that actual durations did not differ largely. It is possible, that shoeing or barefoot status was similar over a much longer time period even in the horses, where a 12-month history was given, as the differences in shape of the distal phalanx documented in the present study are expected to require at least that long to be established, even if rapid changes in hoof biomechanics can be seen immediately after shoeing (11). However, further investigations are necessary to understand the temporal dynamics of distal phalanx morphology exposed to different forces.

The present study revealed notable differences in bone density and shape of the distal phalanges between male and female equids. Analysis of 97 Oxspring radiographs from male equids and 78 Oxspring radiographs from female equids demonstrated that female equids have denser distal phalanges medially and laterally compared to males, while male equids had longer middle phalanges. These findings align with previous research on sex related differences in equine bone density. For instance, a study on Lusitano foals found higher leptin levels in females, which negatively correlated with osteocalcin (92). Leptin has been shown to inhibit osteoclast differentiation in mouse cells and potentially act as a local inhibitor of bone resorption *in vivo* (93). This could explain the higher local bone densities observed in female equids in the present study, potentially due to reduced bone resorption. Additionally, research on Arabian horses has shown sex related differences in phalangeal measurements, with males having longer proximal, middle, and distal phalanges (94). The finding that distal phalanges in male equids are longer compared to females is also observed in the present study. How sex related density and shape changes affect the distal phalanx and shoeing requires further investigations.

## 5 Conclusion

The present study provides novel insights into potential effects of dorsoabaxial clips on the equine distal phalanx. The observed shape differences of the distal phalanx and the trend toward increased bone density in the area of dorsoabaxial clips suggest that these shoeing practices may have more profound effects on hoof biomechanics and bone remodeling than previously recognized. These findings also have important implications for equine medicine. The potential long-term effects of distal phalanx deformation on hoof health locomotion stability and overall equine performance warrant further investigation. Future studies may consider longitudinal designs to track changes over time associated with shoeing differences.

## Data Availability

The data that support the findings of this study are available from the corresponding author upon reasonable request. Requests to access these datasets should be directed to Lisa Henrietta Ennsmann, lisa.henrietta.ennsmann@vetmeduni.ac.at.
