# Correction: Association between radiographic equine distal phalanx characteristics and absence, presence and type of horseshoes

**DOI:** 10.3389/fvets.2025.1675356

**Published:** 2025-08-18

**Authors:** Lisa Henrietta Ennsmann, Theresia Franziska Licka

**Affiliations:** ^1^Department of Companion Animals and Horses, University Clinic for Horses, University of Veterinary Medicine, Vienna, Austria; ^2^Department of Veterinary Clinical Sciences, Large Animal Hospital, Easter Bush Veterinary Centre, Easter Bush, Royal (Dick) School of Veterinary Studies, The University of Edinburgh, Roslin, Midlothian, United Kingdom

**Keywords:** horse, distal phalanx, radiology, horseshoe, clips

In the published article, there was a mistake in the abstract. Within the abstract, the number of warmblood horses displayed was *n* = 137. The correct number is *n* = 132.

A correction has been made to the **abstract**. This sentence previously stated:

“Researchers analyzed Oxspring radiographs of either the left or right front hoof from warmblood horses (*n* = 137) and ponies (*n* = 43) aged 3–28 years”.

The corrected sentence appears below:

“Researchers analyzed Oxspring radiographs of either the left or right front hoof from warmblood horses (*n* = 132) and ponies (*n* = 43) aged 3–28 years”.

The original article has been updated.

